# Review of Burden, Clinical Definitions, and Management of COVID-19 Cases

**DOI:** 10.4269/ajtmh.20-0564

**Published:** 2020-07-01

**Authors:** Laura McArthur, Dhanasekaran Sakthivel, Ricardo Ataide, Felicia Chan, Jack S. Richards, Charles A. Narh

**Affiliations:** 1 School of Medicine, Monash University, Clayton, Australia;; 2 ZiP Diagnostics Pty Ltd, Collingwood, Australia;; 3 Department of Medicine, University of Melbourne, Melbourne, Australia;; 4 Walter and Eliza Hall Institute, Melbourne, Australia;; 5 Central Clinical School, Monash University, Clayton, Australia;; 6 Burnet Institute for Medical Research, Melbourne, Australia

## Abstract

Our understanding of SARS-CoV-2, the virus responsible for coronavirus disease 2019 (COVID-19), its clinical manifestations, and treatment options continues to evolve at an unparalleled pace. This review sought to summarize the key literature regarding transmission, case definitions, clinical management, and the burden of COVID-19. Our review of the literature showed that SARS-CoV-2 was mainly transmitted via inhalation of respiratory droplets containing the virus and had a mean incubation period of 4–6 days. The commonly reported symptoms were fever (75.3% ± 18.7%) and cough (62.6% ± 17.7%) across the spectrum of clinical disease—mild, moderate, severe, and critical, but with the disease phenotype varying with severity. Categorization of these cases for home care or hospital management needs to be defined, with risk stratification accounting for the age of the patient and the presence of underlying comorbidities. The case definitions varied among countries, which could have contributed to the differences in the case fatality rates among affected countries. The severity and risk of death due to COVID-19 was associated with age and underlying comorbidities. Asymptomatic cases, which constitute 40–80% of COVID-19 cases are a considerable threat to control efforts. The presence of fever and cough may be sufficient to warrant COVID-19 testing, but using these symptoms in isolation will miss a proportion of cases. A clear definition of a COVID-19 case is essential for the management, treatment, and tracking of clinical illness, and to inform the quarantine measures and social distancing that can help control the spread of SARS-CoV-2.

## INTRODUCTION

In December 2019, several Health Centers in Wuhan, in the Hubei Province of China, reported a cluster of patients with pneumonia of unknown etiology.^1,2^ Their clinical presentations were similar to those of SARS outbreak that occurred in 2003.^3–5^ COVID-19 is the third coronavirus disease to cause public health outbreaks and has spread more rapidly and widely than SARS and Middle East respiratory syndrome (MERS). COVID-19 is now pandemic, with millions of confirmed cases and several thousands of deaths associated with the disease in 210 countries and territories. This review provides a discussion of the disease transmission, clinical presentations, variability of case definitions, and review of the clinical management.

## BURDEN AND CASE FATALITY OF COVID-19

Since the first cases were recognized in December 2019, SARS-CoV-2 has spread around the world, with cases and fatalities increasing by the thousands daily. While attempting to define the burden and case fatality of the disease, efforts have been complicated by different case definitions and testing procedures, asymptomatic cases that may go unrecognized and the rapidly evolving nature of the pandemic.

Studies of hospitalized patients have reported fatality rates ranging from 1.4% to 18.9%, and as high as 61.5% among those who were critically ill.^6–10^ Case fatality rates were reportedly higher among older adults and the elderly than among young adults and children. Reported rates include 1.0% among adults aged 50–59 years, 3.5% among 60–69 years, 12.8% among 70–79 years, and 20.2% among 80 years or older.^11^ Among critically ill patients, the case fatality is reportedly higher, reaching 50% among adults aged 40–49 years and 87.5–100% among those older than 70 years.^10^ The precise case fatality rate for countries affected by the disease is unknown—although some models allowing for mild and asymptomatic cases estimated it at 0.51%.^12^ Despite this uncertainty, several risk factors for significant outbreaks of severe and fatal illness have been identified. These include patient characteristics, disease phenotype, and laboratory biomarkers.^6,11,13–16^ See Factors associated with COVID-19 morbidity and mortality for factors associated with morbidity and mortality.

## TRANSMISSION ROUTES OF SARS-CoV-2

**Figure 1.**
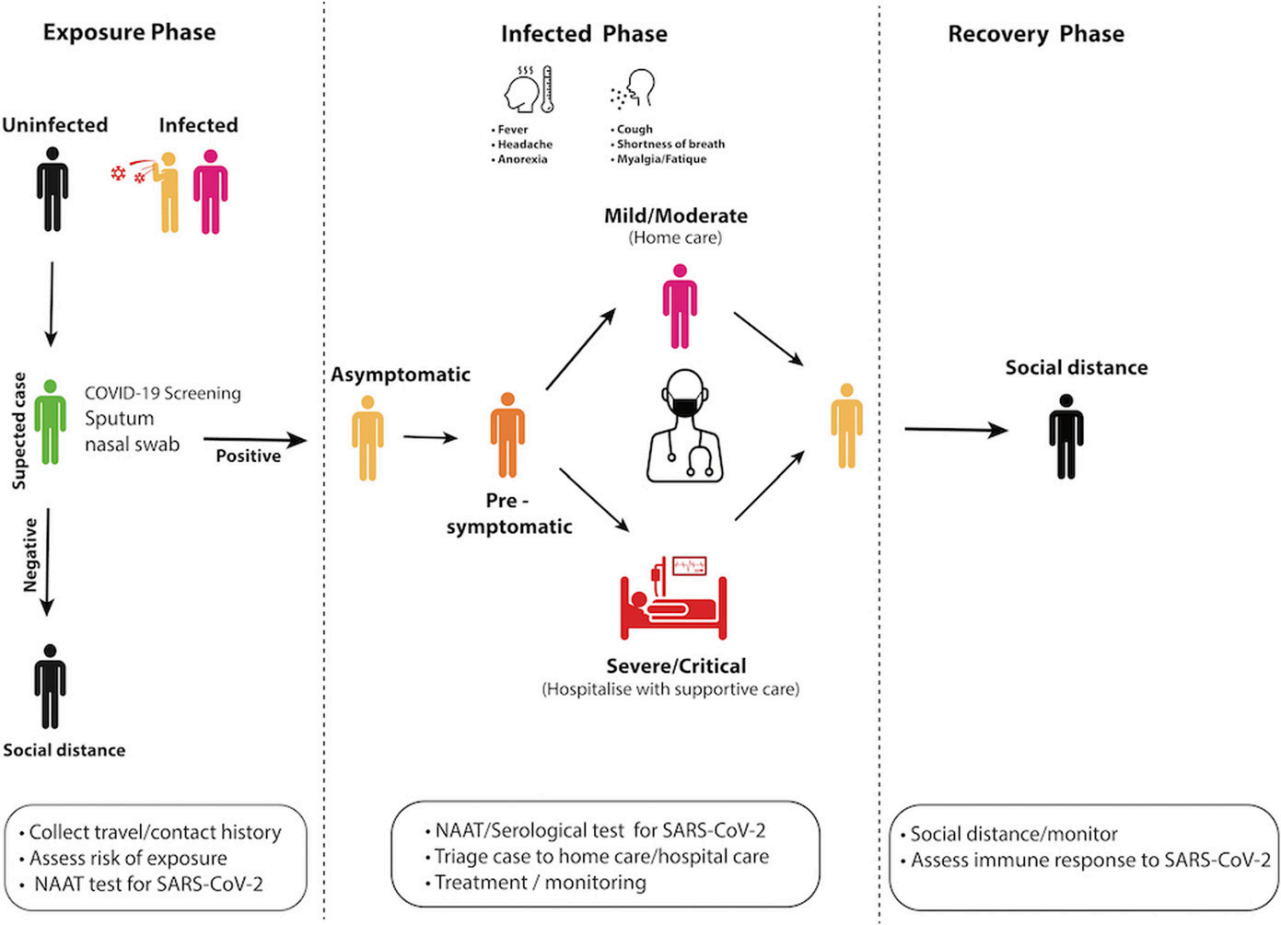
Identification and management of COVID-19 cases. Monitoring for suspected cases of COVID-19 is crucial to halt the transmission of SARS-CoV-2. Suspected cases who have had contact with an infected person (asymptomatic/symptomatic) need to be isolated and screened for SARS-CoV-2 using sensitive nucleic acid amplification tests (NAATs). It is recommended that infected individuals who are asymptomatic self-isolate and be monitored at home. Individuals who progress to develop clinical disease can be triaged into mild/moderate and severe/critical case for clinical management/treatment. However, the presence of comorbidities and the age of the patient can be used to triage patients for hospitalization or home care. Once recovered, patients should be monitored because they could still be infectious.

SARS-CoV-2 is transmitted between humans via respiratory droplets which are produced when an infected individual talks, sneezes, or coughs (Figure 1). Droplet transmission can occur within 1–4 m.^17–19^ SARS-CoV-2 has been shown to survive in aerosolized form for more than 3 hours under experimental conditions, but this mechanical generation of aerosols is unlikely to mimic the true clinical scenario.^20^ Certain clinical procedures involving the upper airway such as obtaining a nose or throat swab, endotracheal intubation, manual ventilation, or nebulization are capable of generating particles < 5 µm, allowing for airborne transmission in healthcare settings.^19^ In particular, intensive care units (ICUs) have been associated with a higher risk of infection.^17^

Fomite transmission, transmission from contact with contaminated surfaces, is possible with high rates of contamination of floors and the soles of healthcare staff as well as computer mice, doorknobs, and trash cans.^17^ The virus is viable for up to 72 hours on plastic and stainless steel, 24 hours on cardboard, and 4 hours on copper. These survival times appear to be longer than those of SARS-CoV-2 under similar conditions and may contribute to the broader spread of SARS-CoV-2 in comparison.^20^

Infection from direct contact with body fluids from infected individuals is likely to be another possible route of transmission. SARS-CoV-2 has been detected in saliva, blood, urine, tears, feces, and cerebrospinal fluid samples.^21–25^ Although documented evidence of transmission through these alternate sources remains unsubstantiated, precautions when handling samples collected from suspected or confirmed cases is advisable.

The basic reproductive number (*R*_0_) of SARS-CoV-2 varies between populations and depends on demographic and environmental factors as well as on the control interventions in place.^26^ At the onset of the outbreak in Wuhan, before travel restrictions were introduced, the estimated *R*_0_ was 2.35 (95% CI: 1.15–4.77) but was reportedly reduced to 1.05 (95% CI: 0.41–2.39) a week later.^27^ Similarly, aboard the *Diamond Princess* cruise ship, which carried ∼3,600 people, 712 cases of COVID-19 and 13 deaths were reported. The *R*_0_ was reported as 14.8, before quarantine/isolation precautions were in place, and was reduced to 1.78 after those measures were introduced.^28^ In closed settings, such as nursing facilities, SARS-CoV-2 may spread rapidly, with one study finding a 64% positivity rate among residents 23 days after the first positive test.^29^ Of these patients, 56% tested positive while still asymptomatic, and it has been hypothesized that asymptomatic individuals contribute significantly to transmission.^29^

## CLINICAL PRESENTATION OF SARS-CoV-2 INFECTIONS

The reported incubation period for SARS-CoV-2 has been variable between studies but has generally ranged between 2 and 11 days, with an average of 4–6 days.^30^ In one study, the incubation period was estimated at 4.9 days (95% CI: 4.4–5.5) and was not significantly different from that of SARS-CoV (4.7, 95% CI: 4.3–5.1) and MERS-CoV (5.8, 95% CI: 5.0–6.5).^31^

**Figure 2.**
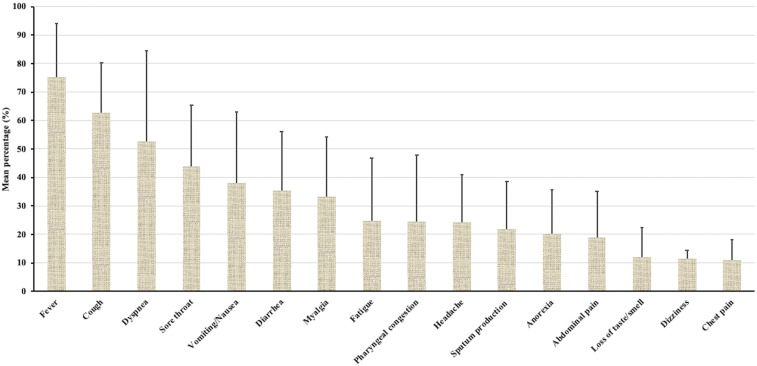
Commonly reported clinical symptoms of COVID-19. Data were obtained from 21 studies involving COVID-19 patients including children and adults. For the pooled analysis, the mean percentage of patients who developed a particular symptom was plotted with the upper standard error. Fever and cough were the commonly reported symptoms. Data were obtained from published data.^6,10,18,32,34,39,57,59–61,68,69,105,124–129^

Symptomatic patients with COVID-19 develop a clinical syndrome similar to that of influenza. Our analysis of 21 studies involving COVID-19 patients showed that the majority of patients with clinical disease presented fever (75.3%), cough (62.6%), dyspnea (52.7%), and sore throat (43.9%) as the commonly reported symptoms (Figure 2). Other reported symptoms were moderately common (20–38%) and included vomiting/nausea, diarrhea, myalgia, fatigue, pharyngeal congestion, headache, sputum production, and anorexia (Figure 2). The less commonly reported symptoms (< 20%) were abdominal pain, loss of taste/smell, dizziness, and chest pain (Figure 2).^1,32–35^ Most of these symptoms appeared within 11 days postinfection, but this may vary depending on age and comorbidities.^6,8,26,36^

Chest X-ray (CXR) and computed tomography (CT) features of disease may appear before COVID-19 disease becomes symptomatic and have been used to aid in diagnosis. On CXR, the most common findings are consolidation and ground-glass opacities, often with bilateral involvement.^37^ On CT, the disease detection rate is high among symptomatic individuals with a meta-analysis study indicating that ground-glass opacities are the most common feature, appearing in 83.31% of cases. This is followed by ground-glass opacities with mixed consolidation in 58.42%, with other common features including adjacent pleural thickening, interlobular septal thickening, and air bronchograms.^38^

Where illness warrants hospitalization, studies suggest that this usually occurs within 5–7 days of symptom onset.^26,39^ The average duration of hospital admission ranges from 7 to 17 days and is dependent on disease severity.^6,40,41^ The WHO recommends 2–6 weeks of hospitalization to allow time for proper treatment and adequate recovery.^42^ The disease can progress from mild to critical, with severe respiratory complications and multiple organ dysfunction, which can be fatal.^1,33,43^ In COVID-related deaths, the median time from symptom onset to death was ∼13–16 days.^44,45^

### Asymptomatic cases.

Asymptomatic SARS-CoV-2 infections, where patients test positive for the virus yet remain clinically well (Figure 1), constitute a large proportion of COVID-19 cases. As such, a population screening strategy based on clinical symptoms alone will miss these cases, increasing transmission risk. Estimates suggest that up to 40–80% of people who test positive for SARS-CoV-2 are asymptomatic.^46,47^ In a proportion of individuals who are initially asymptomatic, a clinical illness will subsequently develop, classifying this group as presymptomatic. A proportion of these patients may have early identifiable pathology with studies of clinically well SARS-CoV-2–positive patients finding that 50–67.3% had lung abnormalities on CT at admission and a proportion of these later developed clinical illness. Among those without initial CT changes, only 11% later developed clinical illness.^13,48^ Presymptomatic cases have also comprised most of the positive results when testing was performed in nursing facilities, with these cases likely contributing to transmission.^29^ Numerous cases of presymptomatic transmission have been reported, and reports from China suggest that 12.6% of cases in China were transmitted asymptomatically.^49^ These findings raised questions as to whether most of the patients who were classified as “presymptomatic” (Figure 1) may have had mild symptoms that went unreported,^50–52^ and what the extent and transmission risk is of true asymptomatic cases.

However, there remains a proportion of patients who will be asymptomatic throughout their illness course. The distribution of asymptomatic cases varies with age, with infected children more likely to be asymptomatic compared to adults.^48,53,54^ While the degree of infectivity of asymptomatic patients compared to those with clinical illness remains uncertain, there are documented cases of transmission from asymptomatic individuals. These individuals have a median of 9.5 days in which they are able to transmit the virus, and such transmissions are able to cause severe COVID-19 disease.^48,51^ Asymptomatic carriers with SARS-CoV-2 infections pose a great threat to COVID-19 control efforts.

### Mild illness.

Mild COVID-19 illness is defined by uncomplicated symptoms, which can be safely managed in the outpatient setting (Figure 1). Although the WHO separates this category into those with and without mild pneumonia, this distinction does not influence management and is difficult to make in patients managed outside of the hospital. For the purposes of this review, all cases suitable for management in an outpatient setting will be considered together.

Mild COVID-19 is perhaps less well understood than more severe disease phenotypes as it is believed that most of such cases do not present for testing. Based on current data, approximately 81% of confirmed SARS-CoV-2 infections are regarded as mild.^22^ However, it is likely that the number of mild cases has been significantly underestimated. During the early period of the disease outbreak in China, it was estimated that 86% of infections went undocumented because of the fact that those infected developed non-severe symptoms and, therefore, did not present for testing.^34^ The study further estimated that undocumented infections were the sources of infection of 79% of all documented infections.^55^

Symptoms of mild COVID-19 are typically those of an upper respiratory tract infection, with atypical presentations being more common in elderly and immunosuppressed individuals.^56^ It appears that fever is less characteristic in mild cases. In patients identified through screening or managed as outpatients, 40.6–55.9% had fever compared with 71.6–98.6% of hospitalized patients.^39,57–60^ Headache, pharyngeal congestion, and disorders of taste and smell were reported with comparative frequency. Malaise, cough, dyspnea, and myalgia were less typical than in severe cases.^39,57,61–63^ Some authorities are widening case definitions to include symptoms such as headache, loss of taste or smell, and pharyngeal congestion as criteria for suspected cases as 59.4% of mild cases are presenting without fever and 41.6% are presenting without cough.^57,64,65^ Although fatalities have been reported among children, they seem more likely to experience a mild illness, and the case fatality rate appears significantly lower than that reported among adults.^66^ As children can still contribute to transmission, considerations for social distancing remain relevant despite the generally comparative mild illness phenotype in this cohort.^8^

### Severe and critical illness.

Severe COVID-19 (Figure 1) is defined by symptoms of significant respiratory distress which in adults are tachypnea ≥ 30 breaths per minute, oxygen saturation ≤ 93%, PaO_2_/FiO_2_ ratio < 300 mmHg, lung infiltrates > 50% within 24–48 hours, or clinical assessment of severe distress.^15,56^ This definition encompasses acute respiratory distress syndrome (ARDS), defined by acute onset, PaO_2_/FiO_2_ ratio, and bilateral infiltrates on CXR.^67^ Through the course of the illness, most patients will develop a fever (77–98.6%) and cough (48.2–76%).^6,13,22,68^ Other common symptoms include myalgia or fatigue, which appear early in the illness and are seen in around 18–32.1% of cases,^68^ sore throat,^69^ and dyspnea.^22^ Fatigue may ultimately occur in up to 69.6% of patients through the course of the illness, but is nonspecific.^13^ Severe COVID-19 appears to be more common in men, with 54.3–68% of hospitalized patients in studies being males.^13,34^ It has likewise been associated with comorbidities; however, severe illness and death can occur in previously young, healthy individuals, including infants.^70,71^

Complications of severe and critical COVID-19 contribute significantly to the morbidity and mortality burden. Acute respiratory distress syndrome is well recognized, developing in 19.6% of hospitalized patients among early studies in China.^33^ Cardiovascular complications are becoming increasingly apparent as more patients reach stages of severe and critical illness, with cardiomyopathy reported in up to 33% of patients and arrhythmias in 16.7%.^33,72^ Other end-organ dysfunction including acute hepatic and kidney injury is also recognized.^72^ In addition, severe disease appears to be associated with a hypercoagulable state with risk of venous thromboembolism in as many as 27% of patients and arterial thrombotic events in 3.7%, leading to risk of pulmonary embolism, stroke, myocardial infarction, and systemic arterial embolism.^73,74^

There is increasing recognition of the existence of central nervous system (CNS) symptoms in COVID-19 presentations, as well as neurological complications which increase in frequency with disease severity. In a study of hospitalized patients, 36.4% had symptoms that were classified as neurological—dizziness (18.8%), headache (13.1%), impaired consciousness (7.5%), acute cerebrovascular disease (2.8%), seizure (0.5%), and ataxia (0.5%).^75^ These were in addition to symptoms relating to peripheral nervous and sensory function.^75^ Central nervous system symptoms were associated with laboratory risk factors for severe disease including lymphocytopenia; 45.5% of severe infections had nervous system manifestations.^75^ Furthermore, there have been reported cases of COVID-19–associated stroke, encephalitis, acute transverse myelitis, perfusion abnormalities on CT, and Guillain–Barre syndrome.^74,76–81^ These included large-vessel strokes in five patients (younger than 50 years) in New York City, United States.^74^ These observations raise concerns about possible neurological effects of the virus, the mechanisms of which are still a cause for speculation.^82^

Critical cases of COVID-19 are defined by respiratory failure requiring mechanical ventilation, and septic shock or organ dysfunction necessitating intensive care.^15^ Critical COVID-19 cases were recorded in 5–6% of SARS-CoV-2 infections during the epidemic phase in China.^15,71^ In other studies of hospitalized patients, the proportion of COVID-19 cases requiring ICU admission ranged from 5% to 40.7%.^6,9,22,39,83^ A need for invasive mechanical ventilation has been reported in 2.3–12.3% of hospitalized patients,^6,39,60^ whereas the requirement for extracorporeal membranous oxygenation (ECMO) occurred in 0.5–3% of patients.^6,34^ Critical COVID-19 has a case fatality rate of 49.0% recorded among those with critical disease in China, and early studies from the United States suggest it is likely to be similarly high.^71,84^

### Recovery from COVID-19.

Once patients recover from COVID-19 (Figure 1), they may remain contagious, as evidenced by case reports of positive reverse transcriptase-polymearse chain reaction (RT-PCR) throat swabs 5–13 days after symptom resolution. This is even present in recovered patients who have registered a previous swab-negative RT-PCR for SARS-CoV-2.^85^ Viral shedding has been observed for up to 37 days in survivors with a median duration of 20 days.^43^ SARS-CoV-2 has also been detected in fecal specimens 18–30 days after illness onset in children,^69^ suggesting that prolonged shedding may still be possible during and after recovery.

Studies of vertical transmission of SARS-CoV-2 remain limited; the low numbers of documented probable cases suggest it is a rare phenomenon where it occurs.^86–89^ Although SARS-CoV-2–specific IgM has been detected in infant sera of COVID-19–positive mothers, it is as yet uncertain whether this represents passive immunization, such as through an altered placenta, or disease transmission with infant immune response.^90^

### Immunity and reinfection with SARS-CoV-2.

Our understanding of the immune responses against SARS-CoV-2 infections is still unfolding as more patient data are analyzed. Seroconversion in infected individuals was observed 1–2 weeks post-symptom onset.^91^ Studies of immune responses in SARS-CoV-2–infected patients have shown increased presence of follicular helper T cells, and activated CD4^+^ and CD8^+^ T cells with the detection of immunoglobulin A, IgM, and IgG against the SARS-CoV-2 spike, nucleocapsid, and envelop proteins.^23,92–95^ There are suggestions that polyclonal antibodies against SARS-CoV-2 may be used as therapeutics for COVID-19 patients; others have suggested convalescent plasma as a source of these antibodies.^96–98^ Data on the breadth, strength, and longevity of these immune responses to protect against disease and reinfection are limited and remain a field to be explored.^99^ However, there seems to be a general lack of long-lived antibody responses against COVID-19 in general.

There is no reliable evidence to suggest that COVID-19 patients who have recovered from the disease can be reinfected. Available data from reinfection studies in monkeys (rhesus macaques) showed that prior infection with SARS-CoV-2 conferred protection against reinfection with the same strain of the virus.^100^ This suggests that immune response to SARS-CoV-2 could protect against reinfection.^101^ Case reports of SARS-CoV-2 PCR-positive results in individuals who were previously PCR negative have not been thoroughly investigated nor confirmed. Others have suggested that this could be because of re-detection of existing infections, which were circulating at low densities.^102^ Thus, detection of the virus following recovery needs to be interpreted with caution as it is more likely to be shedding of the virus from a resolving infection.

## COVID-19 CASE DEFINITIONS

Defining the scope of the COVID-19 pandemic has been challenging because of the need to refine case definitions as the pandemic progressed and as clinical presentations become more clearly understood. These case definitions have been used to determine whom to test and to guide case investigations of possible contacts. By influencing testing algorithms, they have greatly impacted the confirmed test outcomes. As an example, case definitions in China were changed on February 12, 2020, to include clinically diagnosed mild cases, resulting in an increase of > 15,000 cases in a single day.^7^ Thus, developing consistent case definitions, whenever possible, is necessary to track metrics of the disease and its spread.

COVID-19 case definitions have been developed and modified in different jurisdictions according to local circumstances and authorities (Table 1). In Canada, the definition of a probable case has been widened to require only one symptom of illness in addition to an epidemiological risk factor; the breadth of symptoms required for a suspected case was later increased to include general flu-like symptoms, including headache and sore throat.^65^ In Australia, where case definitions are used to determine testing priorities, additional efforts have been devoted to defining high-risk settings such as residential facilities and the nature of a close contact as it relates to in-person interactions and proximity (Table 1). These definitions are highly significant as they determine who receives testing and which patients need to be regarded as at-risk for transmitting the virus. These precautions require resources—including equipment and personnel—such that case definitions must balance capturing possible COVID-19 infections against burdening the healthcare system with individuals with a low probability of infection.

**Table 1:** Case definitions of COVID-19

Case definition	Confirmed case	Suspect case	Probable case	Contact	Other relevant defined terms
WHO^130,131^	A person with laboratory confirmation of SARS-CoV-2 infection regardless of signs/symptoms.	a. Acute respiratory illness (fever and ≥ 1 sign/symptom of respiratory disease) and travel to a region reporting community transmission in 14 days before symptom onset;b. acute respiratory illness and contact with confirmed or probable case in 14 days before symptom onset; orc. severe acute respiratory illness (requiring hospitalization) and the absence of an alternative diagnosis to fully explain the presentation.	a. A suspect case in whom testing is inconclusive orb. a suspect case for whom testing could not be performed.	Experienced any one of the following:	
				a. face-to-face contact with a within 1 m and for more than 15 minutes;b. direct physical contact with a case;c. direct care for a patient with probable or confirmed COVID-19 disease without using proper personal protective equipment; ord. other situations as indicated by local risk assessments.Note: For confirmed asymptomatic cases, the period of contact is measured as 2 days before through the 14 days after the date on which the sample was taken which led to confirmation.	
				Note: This may occur during 2 days before 14 days after symptom onset in a suspected/confirmed case	
Canada^65^	Patient with laboratory-confirmed SARS-CoV-2 infection with the following:a. test performed at a community, hospital, or reference laboratory running a validated assay andb. consists of detection of at least one specific gene target by nucleic acid amplification test assay.	Symptoms that include ≥ 2 of the following:a. fever or signs of fever,b. cough (new or exacerbated chronic),c. sore throat,d. runny nose,e. headache, andf. meets exposure criteria org. had close contact with a probable case of COVID-19.	a. fever (≥ 38°C) and/or new onset/exacerbation of cough;b. meets COVID-19 exposure criteria; andc. laboratory test has been performed but is inconclusive; ora. fever (≥ 38°C) and/or new onset/exacerbation of cough; andb. close contact with a confirmed case of COVID-19; orc. lived in or worked in a closed facility known to be experiencing an outbreak,	Person who provided care for patient;had other similar close physical contact; orlived with or otherwise had close prolonged contact with probable or confirmed case while the case was ill.	Exposure:In 14 days before onset of illness, has the following:a. traveled to an affected area;b. had close contact with a person with acute respiratory illness who has been to an affected area within 14 days of their illness onset;c. had participated in a mass gathering identified as a source of exposure; ord. had laboratory exposure to biological materials containing SARS-CoV-2.
Australia^64^	A person who has the following:a. tests positive to a validated specific SARS-CoV-2 nucleic acid test;b. virus isolated in cell culture, with PCR confirmation using validated method; orc. undergoes seroconversion to or has a significant rise in SARS-CoV-2 neutralizing or IgG antibody level (≥ 4-fold rise in titre).	a. Fever (≥ 37.5°C) or a history of fever (e.g., chills and night sweats) or acute respiratory infection (e.g., cough, shortness of breath, and sore throat) or loss of smell or taste and either of (b) or (c):b. In the 14 days before illness onset has ≥ 1 of the following:i. close contact with confirmed or probable case;ii. international or interstate travel;iii. passengers or crew who have traveled on a cruise ship;iv. healthcare, aged, or residential care workers and staff with direct patient contact; orv. people who have lived in or traveled through a geographically localized area with elevated risk of community transmission; or	a. detection of SARS-CoV-2 neutralizing or IgG antibody;b. has a compatible clinical illness; andc. meet one or more of the epidemiological criteria in (b) or (c) as per suspect case definition.	a. ≥ 15 minutes, cumulative within a week, face-to-face contact with a confirmed or probable case, up to 48 hours before symptom onset in that case orb. sharing of a closed space with a confirmed or probably case for ≥ 2 hours, up to 48 hours before the symptom onset in that case.The definition includes also direct contact of body fluids/laboratory specimens with inadequate PPE, being in the same hospital room during an aerosol-generating procedure without PPE, aircraft passengers within two rows, and crew members as appropriate.An extended definition of “casual contacts” is also available.	High-risk setting: any setting with evidence of a risk for rapid spread and ongoing chains of infection, such as places where people reside in groups or workplace settings where previous outbreaks have shown large-scale amplification. These include but are not limited to the following:a. aged/residential care facility,b. correctional facility,c. detention center, ord. aboriginal rural and remote communities.Within these settings, an outbreak is defined as a single confirmed case in a resident, staff member, or frequent attendee.
		c. hospitalized patients where no other clinical focus of infection or alternate explanation of the illness is evident.			
European Centre for Disease Prevention and Control^132^	Detection of SARS-CoV-2 nucleic acid in a clinical specimen.	No longer used as a term. Replaced with “possible case,” defined by any of cough, fever, shortness of breath, or sudden onset of anosmia, ageusia, or dysgeusia.	a. Radiological evidence showing lesions compatible with COVID-19 orb. ≥ 1 of: cough, fever, shortness of breath, or sudden onset of anosmia, ageusia, or dysgeusia. and one epidemiological criteria (as in the following text):Epidemiological criteria:i. close contact (high-risk contact, see contact definition) with a confirmed case in 14 days before symptom onset or	Contact with a COVID-19 case within 48 hours before symptom onset in that case to 14 days after. High-risk contact, used in the probable case definition, is defined as any of the following:a. having had face-to-face contact with a case, within 2 m for more than 15 minutes;b. having had physical contact with a case;c. having unprotected direct contact with infectious secretions of a case;	–
	
			ii. having been a resident or staff member in a residential institution for vulnerable people where ongoing COVID-19 transmission has been confirmed, in the 14 days before symptom onset.	d. having been in a closed environment with a case for more than 15 minutes (e.g., a closed room);e. in an aircraft, sitting within two seats in any direction of a case, or being a crew member for that area of the craft; orf. a healthcare worker or other person providing care to a COVID-19 case, or laboratory workers handling specimens from a case, without recommended PPE.	
United Kingdom^133^	–	–	–	–	Possible case:
					a. requiring admission to hospital and evidence of pneumonia or ARDS or influenza-like illness or loss of or change in normal sense of taste or smell or
					b. well enough to remain in community with new continuous cough and/or high temperature and/or a loss of or change in normal sense of taste or smell.
USA CDC^134^	Detection of SARS-CoV-2 RNA in a clinical specimen using a molecular amplification test.	–	a. Meet clinical criteria and epidemiological evidence with no confirmed test;	Being within 6 ft of a case for at least 10–30 minutes, depending on the exposure; in healthcare settings, some exposures may need to be only for a few minutes	Clinical criteria:
			b. meet presumptive laboratory evidence and either clinical criteria or epidemiological evidence; or		a. ≥ 2 of the following: fever, chills, rigors, myalgia, headache, sore throat, and new olfactory and taste disorder;
			c. meet vital records criteria with no confirmatory laboratory testing performed for COVID-19.		b. ≥ 1 of cough, shortness of breath, or difficulty breathing; or
			Epidemiological linkage: ≥ 1 of the following in the 14 days before onset of symptoms:		c. severe respiratory illness with either clinical/radiological evidence of pneumonia or ARDS; and
			a. close contact with a confirmed or probable case;		d. no alternative diagnosis more likely.
			b. close contact with a person with clinical compatible illness and linkage to a confirmed case;		Presumptive laboratory evidence:
			c. travel to or residence in an area with sustained, ongoing community transmission of SARS-CoV-2; or		a. detection of specific antigen in a clinical specimen or
			d. member of a risk cohort as defined by public health authorities during an outbreak.		b. detection of specific antibody in serum, plasma or whole blood indicative of a new or recent infection.
					Vital records criteria:
					a. a death certificate that lists COVID-19 disease or SARS-CoV-2 as a cause of death or a significant condition contributing to death.
China NHC^135^	Suspect cases with ≥ 1 of the following:	Considers the following:	–	–	–
	a. RT fluorescent PCR positive for nCoV;	a. ≥ 1 of the following:			
	b. viral gene sequence highly homologous for nCoV; or	i. history of or travel to Wuhan/surrounds or communities with cases within 14 days;			
	c. virus-specific IgM and IgG detectable in serum, with IgG at least 4-fold increase during convalescence.	ii. in contact with nCoV-infected people within 14 days;			
		iii. in contact with patients with fever/respiratory symptoms from regions with confirmed cases; and			
		iv. clustered cases (≥ 2 with symptoms, e.g., in family, office, or school) and			
		b. ≥ 2 of (≥ 3 if failing to meet (a) above) the following:			
		i. fever and/or respiratory symptoms;			
		ii. imaging characteristics; and			
		iii. normal or decreased WCC, and normal or decreased lymphocytes in early stages.			
NICD, South Africa^136^	Laboratory-confirmed infection with SARS-CoV-2.	Defined as PUI.	PUI for whom SARS-CoV-2 testing is inconclusive or	A person having the following:	–
		a. Acute respiratory illness (≥ 1 of fever (or history of fever), cough, sore throat, and shortness of breath) and	tested positive in a pan-COVID-19 assay.	a. face-to-face contact (≤ 2 m) or being in a closed environment with a COVID-19 case;	
		b. close contact with a confirmed or probable case;		b. HCW/person providing care while not wearing recommended PPE; or	
		c. history of travel to area with local transmission;		c. within two seats of COVID-19 patient on an aircraft, or crew members for that section.	
		d. worked in or attended a healthcare facility where COVID-19 patients are being treated; or			
		e. admitted with severe pneumonia of unknown aetiology.			

ARDS = acute respiratory distress syndrome; PPE = personal protective equipment; PUI = person under investigation; WCC = white cell count.

## FACTORS ASSOCIATED WITH COVID-19 MORBIDITY AND MORTALITY

The risk of severe or fatal COVID-19 has been associated with three key categories of risk factors: patient characteristics, disease characteristics, and biomarkers.

### Patient characteristics.

Age has consistently been associated with risk of COVID-19 disease. Among COVID-19 patients, older adults and the elderly compared with young adults and children have had an increased likelihood of severe disease, increased risk of mortality, higher admission rates, and increased length of hospital stay.^9,11,16,41,43,60,103,104^

COVID-19 mortality and morbidity have further been associated with comorbidities (Table 2), which may vary between countries.^6,68^ In the general population, the mortality rate (2.3%) due to COVID-19 was reportedly lower than among patients with chronic disease, increasing to 10.5% in patients with cardiovascular disease, 7.3% among patients with diabetes mellitus, 6.3% among those with chronic respiratory diseases, 6% among patients with hypertension, and 5.6% in cancer patients.^71^ Such diseases also increase the risk of requiring invasive ventilation, with this reported at increased rates among patients with obesity, diabetes, hypertension, chronic pulmonary disease, cardiovascular disease, and cancer.^16,41,105,106^ In the United States, underlying health conditions and risk factors were identified in 27% of nonhospitalized, 71% of hospitalized, and 78% ICU cases due to COVID-19.^107^ In one Italian study, 99.2% of COVID-19 cases admitted to the hospital had underlying chronic diseases, and in China, chronic illness was identified in 40% of cases of critical illness.^10,11^

**Table 2:** Risk factors for fatal disease

	HR	OR
Patient comorbidities		
Chronic cardiac disease	1.16–1.76	–
Chronic pulmonary disease	1.17–2.94	–
Chronic kidney disease	1.28	–
Obesity	1.33	–
Chronic neurological disorder	1.17	–
Dementia	1.40	–
Malignancy	1.13–1.3	–
Liver disease	1.51	–
Disease characteristics at presentation		
Oxygen saturation < 88%	2.0	–
SOFA score	–	5.65
Biomarkers		
Raised C-reactive protein	> 3.5	–
Raised initial D-dimer	1.02–2.2	18.42
Elevated troponin	2.1	–
Neutrophilia	1.08	–
Elevated lactate dehydrogenase	1.30	–
Elevated interleukin 6	1.11 (per decile increase)	–

HR = hazard ratio; OR = odds ratio.

Hazard ratio and OR were obtained from the reported data: patient comorbidities,^10,41,43,60,84,112^ disease characteristics,^10,41,43,45,105^ and biomarkers.^10,41,43,45,75,104,105,112^

Other risk factors including gender, host genetics, and country of residence have been reported. Most of the patients with severe and fatal disease across multiple settings have been male, although the reasons for this remain unclear.^41,45,60^ In addition, factors including ethnicity, health insurance status, and country of residence have been implicated as possible risk factors for disease outcome.^108^ Host genetics including polymorphisms in the human receptor, angiotensin-converting enzyme-2, for SARS-CoV-2, may play a role in infection and severity of COVID-19.^109–111^

### Disease characteristics.

Several disease characteristics at presentation have additionally been identified as markers of progression from mild/moderate to severe disease. These have been previously outlined in terms of disease phenotype for mild versus severe/critical disease. They include fever, dyspnea, tachypnea, and chest tightness.^41,45,105^ Higher Sequential Organ Failure Assessment (SOFA) scores have been associated with increased mortality risk and may be a tool for assessing mortality risk at admission.^10,43^

### Biomarkers.

Biomarkers including neutrophilia, lymphocytopenia, and elevated creatinine, bilirubin, aspartate aminotransferase, troponin, D-dimer, interleukin 6, ferritin, C-reactive protein, lactate dehydrogenase, blood urea nitrogen, creatinine kinase, and procalcitonin have been associated with severe disease and mortality due to COVID-19.^10,41,43,45,75,104,105,112^

## MANAGEMENT OF COVID-19 CASES

For most patients, COVID-19 presents as a mild illness that can be managed at home with rest and simple analgesics/antipyretics for symptom relief (Figure 1). Paracetamol has been suggested as the drug of choice for symptom relief, whereas anecdotal evidence of non-steroidal anti-inflammatory drug (NSAID)-associated harm in COVID-19–infected patients is being investigated.^113^

To not overburden health staff, most healthcare facilities manage milder cases of COVID-19 on an outpatient basis, with patients self-isolating in their own homes. As yet, the benefits of early interventions that have been proposed—such as oxygen therapy—remain unclear.^114^ The rapidly rising case numbers in many countries continue to impose a significant burden on the healthcare system, making inpatient management of mild cases challenging.

Once patients are admitted to hospital, treatments fall into three key categories: supportive care, treatment of coinfection and comorbidity, and disease-modifying treatments, which currently remain experimental.

For patients with severe or critical SARS-CoV-2, supportive care is the current mainstay of treatment (Figure 1). This includes attention to fluids and electrolytes, monitoring for complications, facilitating symptomatic management, and providing respiratory support. Respiratory support can be provided in a stepwise fashion as required, moving from oxygen therapy through to noninvasive ventilation, and then intubation and mechanical ventilation. In ARDS, there is evidence that prone positioning of patients may improve oxygenation, and it is currently being recommended for hospitalized COVID-19 patients.^64,66,115^ With a high fatality rate observed in mechanically ventilated patients and pandemic cases placing enormous strain on global ventilator supply, it is of note that isolated hypoxemia can be well tolerated where respiratory effort remains in an acceptable range and is not necessarily an appropriate trigger for intubation.^115^ Where intubation and mechanical ventilation is required, it should be tailored to the disease phenotype and aim to prevent further lung injury.^115^ Extracorporeal membranous oxygenation may be used if available for refractory hypoxia. It is important to note that although respiratory support measures are integral to COVID-19 management, they also create high-risk environments in which airborne transmission of the virus may be possible.^19^ These measures include intubation, nebulized treatments, moving patients to a prone position, and positive-pressure noninvasive ventilation. During these activities, it important that healthcare personnel wear appropriate personal protective equipment including N95 mask and eye protection (Figure 1).

Where COVID-19 is causing severe illness or sepsis, the WHO recommends empirical antimicrobial treatment, with other sources suggesting this be considered in any severe infection.^66^ In addition, consideration may be given to a neuraminidase inhibitor in the event of coinfection with influenza. Coinfection of COVID-19 patients with other pathogens including influenza A and B, respiratory syncytial virus, rhinovirus, and adenovirus may be common, occurring in 22% of cases in some reports.^116^ However, this depends on region and season. Coinfection with other respiratory pathogens may increase COVID-19 severity. As such, severe disease warrants testing and treatment for these coinfections.

Disease-modifying treatments for COVID-19 remain under investigation (Supplemental Tables 1 and 2). Of note in this category, preliminary results from the Recovery trial indicate that low-dose dexamethasone reduces mortality among COVID-19 patients requiring respiratory support, by up to one-third in those requiring ventilation.^117^ As such, and with no significant harms associated with the medication in this trial, dexamethasone is now being recommended for consideration in severe disease.^118^ In addition, there is ongoing interest in remdesivir, an antiviral previously trialed in Ebola virus disease. Whereas early trials were equivocal regarding possible benefit,^119^ recent preliminary results from a randomized controlled trial suggest remdesivir may improve recovery time and rate from COVID-19.^120^ There are numerous other drugs under ongoing investigation because of their potential to modify some aspect of the COVID-19 disease course (Supplemental Table 1); these have been reviewed elsewhere and remain an area of active research.^121,122^ Meanwhile, there is extensive ongoing research into potential candidate vaccines (Supplemental Table 2), with 13 in clinical evaluation and hundreds more being studied. Candidates include RNA, DNA, protein subunit, nonreplicating viral vector, and inactivated platforms. These additions will continue to be of great significance as control efforts continue.^123^ See Supplemental Table 1 for drugs and Supplemental Table 2 for vaccines and immunotherapies in development for COVID-19.

## CONCLUSION

SARS-CoV-2, the causative agent of the novel coronavirus disease, COVID-19, has caused a huge disease burden globally in the short time it has been recorded. A clear description of symptoms associated with the disease is crucial to establish the case definition for clinical management and for epidemiological purposes. Most of the patients are likely to remain asymptomatic or mildly symptomatic. Those that do develop clinical disease will most typically have fever and cough, but these remain absent in up to 59.4% and 41.6% of cases, respectively, creating challenges for disease detection and control. The presence of either of these symptoms should be a basis for suspecting a SARS-CoV-2 infection. However, because other respiratory viruses present with similar symptoms, a laboratory test specific to SARS-CoV-2 should be performed.

Nearly every country in the world has been affected by COVID-19. It is important to note that the definitions for COVID-19 cases vary between countries and territories affected by the disease, and this in turn may have affected the public health response. When defining cases, it is important that national guidelines account for the age distribution of the population and the presence of comorbidities including cardiovascular diseases, diabetes, and cancer, which increase the risk of developing severe and/or critical disease, and increase the risk of fatality. Where transmission is still in the exponential phase of SARS-CoV-2 infections, it is critical that clinical cases are triaged to prioritize management and treatment without overwhelming the healthcare system.

Although several drugs are beginning to show promise in clinical trials, it may take months before these become sufficiently studied and available for widespread clinical use. Hence, it is important that quarantine and isolation measures are strictly enforced to control the disease outbreak. A major challenge, however, to controlling SARS-CoV-2 transmission is how to identify the “silent spreaders” who are asymptomatic carriers of the infection.

### Limitations of the study.

During the review period, the data on COVID-19 constantly changed with increasing amounts of literature, both peer-reviewed and non–peer-reviewed. COVID-19 data were dependent on country-level definitions and testing rates.

## Supplemental tables

Supplemental materials
