# Supplementary material for: Review of Burden, Clinical Definitions, and Management of COVID-19 Cases
Source: Am J Trop Med Hyg. 2020 Jul 1;103(2):625–38. doi: 10.4269/ajtmh.20-0564 (PMC7410412; doi:10.4269/ajtmh.20-0564)
Supplement: Supplementary file 1 [file tpmd200564.SD1.pdf]

Table S1: Current drugs and therapies in development for COVID-19

Drug/treatment	Description/mode of action	Clinical trial stage	Manufacturer	Comments	Reference
Chloroquine / Hydroxychloroquine	Inhibits SARS-CoV-2 replication and prevent the onset of acute respiratory distress syndrome (ARDS) as it stimulates an anti-inflammatory response.	US FDA has revoked its approval for use in COVID-19 due to no significant benefit in early clinical trials	Novartis, Mylan, Teva	No significant association between the use of hydroxychloroquine and recovery. Both drugs have been associated with negative side-effects.	[1] [2] [3] [4]
Lopinavir / Ritonavir	Lopinavir is a viral protease inhibitor, helps in suppressing viral replication. Ritonavir is also a viral protease inhibitor but mainly serve to enhance the concentration of lopinavir	Clinical trial	Abbott Laboratories (FDA approved in 2000)	Lopinavir and ritonavir is currently used in combination to treat Human immunodeficiency virus (HIV) and have been identified to be effective in treating MERS-CoV infections in animal models.	[5] [6] [7]
Remdesivir	Block viral replication by acting as a competitive inhibitor to RNA-dependent RNA polymerase	Phase 3; US FDA Emergency use authorisation for severe COVID-19	Gilead Sciences	Broad spectrum antiviral drug tested on MERS-CoV and SARS-CoV but not on SARS-CoV-2. US FDA has issued Emergency use authorisation for severe COVID-19.	[8] [9] [10]
Gimsilumab	Monoclonal antibody against GM-CSF protein, helps reduce inflammation to treat and/or prevent the onset of ARDS	Phase 3	Roivant Sciences	Blocking GM-CSF inhibits downstream signalling pathways, preventing the onset of cytokine storm	[11] [12]

MultiStem	Stem-cell therapy used to treat ARDS by harvesting stem cell from adult bone marrow	Phase 2	Athersys	Stem cell therapy facilitates in clearing the virus through apoptosis and phagocytosis. Steam cell therapy also releases mediators such as anti-inflammatory cytokines, antimicrobial peptides and restore protein permeability of endothelial cells, preventing secondary microbial infection and treating lung damage	[13] [14] [15] [16] [17]
Danoprevir and Ritonavir	Danoprevir is a protease inhibitor which has high affinity binding to SARS-CoV-2 protease, preventing the cleavage of polyprotein that forms the replicase-transcriptase complex. Ritaonavir is also a protease inhibitor, but mainly serves to enhance the concentration of danoprevir	Phase 1	Ascletis Pharma	Antiviral combination therapy, danoprevir used to treat Hepatitis C and Ritonavir used to treat HIV. Protease function in both Hepatitis C and HIV have proteases similar to SARS-CoV-2 protease, hypothesised that these drugs will inhibit SARS-CoV-2 protease function.	[18] [19]
CYNK-001	Generate natural killer cells through steam cells differentiation	Phase 1	Celularity	Natural killer cells are essential in killing virally infected cell, facilitating in viral elimination	[20] [21]
TJM2	Formation of antibody against GM-CSF to reduce inflammation and sequential ARDS onset	Phase 1	I-Mab		[22]
EIDD-2801	Block viral replication by acting as a ribonucleaside analogue	Phase 1	Ridgeback Biotherapeutics	Administered orally	[23]

SAB-185	Polyclonal antibodies allowing more than one viral protein target	Preclinical	SAB Biotherapeutics and CSL Behring	Treatment using humanised polyclonal antibodies, without the need for serum therapy from COVID-19 survivors	[24]
	In-vitro CoV activated T cells to transplant into patients to facilitate in eliminating active CoV infection	Preclinical	Allovir	Harvesting T cells from healthy donors and activating them by incubating it with harmless fragments of CoV	[25]
	Antibody-based treatment	Preclinical	Amgen	Isolate neutralising CoV antibodies from survivors and screen them to identify the most effective ones to use for the develop potential treatment	[26] [27]
	Antibody-based treatment	Preclinical	AstraZeneca and Vanderbilit University Medical Center	Isolating B cells from the serum of COVID-19 survivors to clone and use it for potential treatments. Neutralising antibodies are also being analysed for clinical treatment	[28] [27]
	Antibody-based treatment	Preclinical	Celltrion	Consolidated a library of antibodies obtained from recovered patients and determine which is the most effective in neutralising SARS-CoV-2. Trying to also develop a super antibody that is protective against all viruses that falls within the CoV family	[29] [27]
LY3127804	Monoclonal antibody against angiopoietin 2 (Ang2), to prevent the onset of ARDS and the need for medical ventilators	Preclinical	Eli Lily	Blocking Ang2 reduces lung injury	[30] [31]

	Antibody-based treatment	Preclinical	Grifols	Obtaining plasma sample from COVID-19 survivor and using it for potential COVID-19 treatment	[32] [27]
	Drug that generates antibodies (through mRNA sequencing) with these antibodies localising within the lungs	Preclinical	Neurimmune and Ethris	Ethris working on getting anti-CoV antibodies directly into the lungs of infected individuals via inhaler and Neurimmune trying to identify neutralising antibodies from COVID-19 survivors.	[33] [27]
	Block viral replication by targeting viral replication enzyme	Preclinical	Pfizer	Similar compounds Pfizer used previously to develop SARS-CoV antiviral medication	[34]
	Antibody-based treatment	Preclinical	Regeneron Pharmaceuticals	Formation of SARS-CoV-2 antibodies through infecting mice with harmless analogs of SARS-CoV-2	[35]
CoVIg-19	Antibody-based treatment	Preclinical	Takeda, CSL Behring, Biotest, BPL, LFB and Octapharma	Obtaining plasma sample from COVID-19 survivors and isolating protective antibodies.	[36] [27]
	Block viral replication	Preclinical	Vir Biotechnology and GlaxoSmithKline (GSK)		[37]

Table S2: Current vaccines in development for COVID-19

Vaccine	Description/mode of action	Clinical trial stage	Manufacturer	Comments	Reference
Ad5-nCoV	Obtaining genetic fragments of SARS-CoV-2 and encoding it within adenovirus type -5 (Ad5) vector	Phase 2	CanSino Biologics	Stimulates the adaptive immune response resulting in the formation of SARS-CoV-2 protective antibodies	[38]
mRNA-1273	Synthetic mRNA that transcribes for the spike of SARS-CoV-2	Phase 2	Moderna Therapeutics		[39]
BNT162	Uses specific genomic sequence of SARS-CoV-2 mRNA	Phase 2	BioNTech and Pfizer		[40] [41]
LV-SMENP-DC	Uses lentiviral vector to modify dendritic cells to express specific viral proteins which is sequentially used to activate antigen specific CD8 T cells	Phase 2	Shenzhen Geno-Immune Medical Institute	Lentiviral dendritic cell and CD8 T cell vaccine	[42]
ChAdOx1 nCoV-19	Integrating the genomic sequence of SARS-CoV-2 spike protein into an adenovirus vaccine vector	Phase 1/2	University of Oxford		[43]
	Uses lentiviral vector to express specific viral proteins on artificial antigen presenting cells	Phase 1	Shenzhen Geno-Immune Medical Institute	Artificial antigen presenting cell vaccine	[44]
INO-4800	Delivering plasmids encoding for the DNA into cells, allowing the generation of SARS-CoV-2 protective antibodies	Phase 1	Inovio Pharmaceuticals	DNA vaccine	[45] [46]
	Identifying the genomic sequence of SARS-CoV-2 that gives rise to immunity and integrating the genomic	Preclinical	Johnson & Johnson		[47]

	sequence into the genome of a harmless virus				
LUNAR-COV19	Identify the genomic sequence of SARS-CoV-2 that gives rise to immunity and integrate the genomic sequence into an RNA virus before delivering it using nanoparticle	Preclinical	Arcturus Therapeutics and Duke-NUS	Nanoparticle stimulates the expression of viral antigen on both MHC class I and class II as it has the ability to enter antigen-presenting cells through different pathways, this allows good immune response with low doses.	48<br>21
COVID-19 S-Trimer	Formulating vaccine consisting of SARS-CoV-2 surface proteins and adjuvants	Preclinical	Clover Biopharmaceuticals and GSK	Protein-based vaccine	49
	Man-made mRNA sequence that translates into viral proteins	Preclinical	CureVac	mRNA-based vaccines	50
NVX-CoV2373	Formulating vaccine consisting of SARS-CoV-2 and Matrix-M adjuvants	Preclinical	Novavax	Animal models identified high levels of neutralising antibodies towards SARS-CoV-2 spike proteins	51
	Adjuvant vaccine for SARS-CoV-2	Preclinical	Sanofi and GSK	Used recombinant DNA technology to produce identical spike proteins expressed on SARS-CoV-2	52
	Protein vaccine that contains SARS-CoV-2 key protein	Preclinical	University of Queensland and GSK	Used molecular clamp technique that locks the natural shape of SARS-CoV-2 spike protein	53

References

1. Jaffe S, 2020. Regulators split on antimalarials for COVID-19. The Lancet 395: 1179.
2. Principi N, Esposito S, 2020. Chloroquine or hydroxychloroquine for prophylaxis of COVID-19. The Lancet Infectious Diseases.

3. 2020. Coronavirus (COVID-19) Update: FDA Revokes Emergency Use Authorization for Chloroquine and Hydroxychloroquine. Available at: https://www.fda.gov/news-events/press-announcements/coronavirus-covid-19-update-fda-revokes-emergency-use-authorization-chloroquine-and. Accessed 24/06/2020, 2020.
4. Geleris J*, et al.*, 2020. Observational Study of Hydroxychloroquine in Hospitalized Patients with Covid-19. New England Journal of Medicine 382: 2411-2418.
5. Patick AK, Potts KE, 1998. Protease inhibitors as antiviral agents. Clinical microbiology reviews 11: 614-627.
6. Molla A, Mo H, Vasavanonda S, Han L, Lin CT, Hsu A, Kempf DJ, 2002. In Vitro Antiviral Interaction of Lopinavir with Other Protease Inhibitors. Antimicrobial Agents and Chemotherapy 46: 2249.
7. Cao B*, et al.*, 2020. A Trial of Lopinavir–Ritonavir in Adults Hospitalized with Severe Covid-19. New England Journal of Medicine 382: 1787-1799.
8. 2020. Remdesivir Clinical Trials. Available at: https://www.gilead.com/purpose/advancing-global-health/covid-19/remdesivir-clinical-trials. Accessed 28/04/2020, 2020.
9. Gordon CJ, Tchesnokov EP, Feng JY, Porter DP, Gotte M, 2020. The antiviral compound remdesivir potently inhibits RNA-dependent RNA polymerase from Middle East respiratory syndrome coronavirus. J Biol Chem 295: 4773-4779.
10. 2020. Coronavirus (COVID-19) Update: FDA Issues Emergency Use Authorization for Potential COVID-19 Treatment. Available at: https://www.fda.gov/news-events/press-announcements/coronavirus-covid-19-update-fda-issues-emergency-use-authorization-potential-covid-19-treatment. Accessed 24/06/2020, 2020.
11. 2020. Roivant announces development of anti-GM-CSF monoclonal antibody to prevent and treat acute respiratory distress syndrome (ARDS) in patients with COVID-19. Available at: https://roivant.com/roivant-announces-development-of-anti-gm-csf-monoclonal-antibody-to-prevent-and-treat-acute-respiratory-distress-syndrome-ards-in-patients-with-covid-19/. Accessed 28/04/2020, 2020.
12. Vijayvargiya P, Garrigos ZE, Almeida M, Gurram PR, Stevens RW, Razonable M, Razonable RR, 2020. Treatment Considerations for COVID-19: A Critical Review of the Evidence (or Lack Thereof). Mayo Clin Proc 95.
13. 2020. Acute Respiratory Distress Syndrome (ARDS). Available at: https://www.athersys.com/clinical-trials/ards/default.aspx. Accessed 29/04/2020, 2020.
14. Khoury M, Cuenca J, Cruz FF, Figueroa FE, Rocco PR, Weiss DJ, 2020. Current Status of Cell-Based Therapies for Respiratory Virus Infections: Applicability to COVID-19. European Respiratory Journal.
15. Lee RH, Pulin AA, Seo MJ, Kota DJ, Ylostalo J, Larson BL, Semprun-Prieto L, Delafontaine P, Prockop DJ, 2009. Intravenous hMSCs improve myocardial infarction in mice because cells embolized in lung are activated to secrete the anti-inflammatory protein TSG-6. Cell stem cell 5: 54-63.
16. Krasnodembskaya A, Song Y, Fang X, Gupta N, Serikov V, Lee J-W, Matthay MA, 2010. Antibacterial effect of human mesenchymal stem cells is mediated in part from secretion of the antimicrobial peptide LL-37. Stem cells (Dayton, Ohio) 28: 2229-2238.
17. Hu S, Park J, Liu A, Lee J, Zhang X, Hao Q, Lee J-W, 2018. Mesenchymal Stem Cell Microvesicles Restore Protein Permeability Across Primary Cultures of Injured Human Lung Microvascular Endothelial Cells. Stem cells translational medicine 7: 615-624.
18. 2020. Evaluation of Ganovo (Danoprevir) combined with Ritonavir in the treatment of SARS-CoV-2 infection Available at: https://clinicaltrials.gov/ct2/show/NCT04291729. Accessed 28/04/2020, 2020.

19. Chen H, Zhang Z, Wang L, Huang Z, Gong F, Li X, Chen Y, Wu JJ, 2020. First Clinical Study Using HCV Protease Inhibitor Danoprevir to Treat Naive and Experienced COVID-19 Patients. medRxiv: 2020.03.22.20034041.
20. 2020. Sorrento to provide manufacturing support to Celularity as CYNK-001 NK cell trial for COVID-19 begins enrolling patients Available at: http://www.globenewswire.com/news-release/2020/04/02/2010998/0/en/SORRENTO-TO-PROVIDE-MANUFACTURING-SUPPORT-TO-CELULARITY-AS-CYNK-001-NK-CELL-TRIAL-FOR-COVID-19-BEGINS-ENROLLING-PATIENTS.html. Accessed.
21. Luevano M, Madrigal A, Saudemont A, 2012. Generation of natural killer cells from hematopoietic stem cells in vitro for immunotherapy. Cellular & molecular immunology 9: 310-320.
22. 2020. I-Mab to develop therapy for Covid-19-related cytokine storm. Available at: https://www.pharmaceutical-technology.com/news/i-mab-covid-19-cytokine-storm-therapy/. Accessed 28/04/2020, 2020.
23. 2020. Ridgeback Biotherapeutics and DRIVE to develop Covid-19 drug. Available at: https://www.pharmaceutical-technology.com/news/ridgeback-biotherapeutics-covid-19/. Accessed 28/04/2020, 2020.
24. 2020. CSL Behring and SAB Biotherapeutics join forces to deliver new potential COVID-19 therapeutic Available at: https://www.csl.com/news/2020/sab-covid-19. Accessed 28/04/2020, 2020.
25. 2020. AlloVir expands its research collaboration with Baylor College of Medicine to discover and develop allogeneic, off-the-shelf, virus-specific T-cell therapies for COVID-19. Available at: https://www.allovir.com/news-blog/allovir-expands-its-research-collaboration-with-baylor-college-of-medicine-to-discover-and-develop-allogeneic-off-the-shelf-virus-specific-t-cell-therapies-for-covid-19. Accessed 28/04/2020, 2020.
26. Cross R, 2020. Amgen and Adaptive pursue COVID-19 antibody therapy. Available at: https://cen.acs.org/pharmaceuticals/biologics/Amgen-Adaptive-pursue-COVID-19/98/i14. Accessed 28/04/2020, 2020.
27. Piro A, Tagarelli A, Tagarelli G, Lagonia P, Quattrone A, 2008. Paul Ehrlich: The Nobel Prize in Physiology or Medicine 1908. International Reviews of Immunology 27: 1-17.
28. Snyder B, 2020. Vanderbilt University Medical Center and AstraZeneca join forces to identify potential COVID-19 treatments. Available at: https://news.vumc.org/2020/04/08/vanderbilt-university-medical-center-and-astrazeneca-join-forces-to-identify-potential-covid-19-treatments/. Accessed 28/04/2020, 2020.
29. 2020. Celltrion identifies antibody candidates against COVID-19. Available at: https://www.pharmaceutical-technology.com/news/celltrion-identifies-covid-19-antibodies/. Accessed 28/04/2020, 2020.
30. 2020. Lilly Begins Clinical Testing of Therapies for COVID-19. Available at: https://investor.lilly.com/news-releases/news-release-details/lilly-begins-clinical-testing-therapies-covid-19. Accessed 28/04/2020, 2020.
31. Li G*, et al.*, 2020. Assessing ACE2 expression patterns in lung tissues in the pathogenesis of COVID-19. Journal of autoimmunity: 102463-102463.
32. 2020. Grifols Announces Formal Collaboration with US Government to Produce the First Treatment Specifically Targeting COVID-19. Available at: https://www.grifols.com/en/view-news/-/news/grifols-announces-formal-collaboration-with-us-government-to-produce-the-first-treatment-specifically-targeting-covid-19. Accessed 28/04/2020, 2020.

33. 2020. Neurimmune and Ethris Sign Collaboration Agreement to Rapidly Develop Inhaled mRNA-based Antibody Therapy for the Treatment of Covid-19. Available at: https://www.neurimmune.com/news/neurimmune-and-ethris-sign-collaboration-agreement-to-rapidly-develop-inhaled-mrna-based-antibody-therapy-for-the-treatment-of-covid-19. Accessed 28/04/2020, 2020.
34. 2020. Pfizer advances battle against COVID-19 on multiple fronts Available at: https://www.pfizer.com/news/press-release/press-release-detail/pfizer_advances_battle_against_covid_19_on_multiple_fronts. Accessed 28/04/2020, 2020.
35. 2020. Regeneron's COVID-19 response efforts. Available at: https://www.regeneron.com/covid19. Accessed 28/04/2020, 2020.
36. 2020. COVID-19: Working Together to Go Faster for Patients. Available at: https://www.takeda.com/newsroom/featured-topics/working-together-to-go-faster-for-patients/. Accessed 28/04/2020, 2020.
37. Idrus AA, 2020. GSK becomes Vir's newest partner on COVID-19 treatments, vaccines. Available at: https://www.fiercebiotech.com/biotech/gsk-becomes-vir-s-newest-partner-covid-19-treatments-vaccines. Accessed 28/04/2020, 2020.
38. Keown A, 2020. China's CanSino Prepares to Advance COVID-19 Vaccine Candidate into Phase II. Available at: https://www.biospace.com/article/china-s-cansino-prepares-to-advance-covid-19-vaccine-candidate-into-phase-ii/. Accessed 28/04/2020, 2020.
39. Taylor NP, 2020. Moderna's COVID-19 vaccine trial starts enrolling high-dose arm. Available at: https://www.fiercebiotech.com/biotech/moderna-s-covid-19-vaccine-trial-starts-enrolling-high-dose-arm. Accessed 28/04/2020, 2020.
40. 2020. BioNTech and Pfizer get German approval for Covid-19 vaccine trial. Available at: https://www.clinicaltrialsarena.com/news/biontech-pfizer-covid-19-vaccine-trial/. Accessed 28/04/2020, 2020.
41. 2020. A Trial Investigating the Safety and Effects of Four BNT162 Vaccines Against COVID-2019 in Healthy Adults. Available at: https://clinicaltrials.gov/ct2/show/NCT04380701. Accessed 24/06/2020, 2020.
42. 2020. Immunity and Safety of Covid-19 Synthetic Minigene Vaccine. Available at: https://clinicaltrials.gov/ct2/show/NCT04276896. Accessed 24/06/2020, 2020.
43. 2020. Oxford COVID-19 vaccine begins human trial stage. Available at: https://www.research.ox.ac.uk/Article/2020-04-23-oxford-covid-19-vaccine-begins-human-trial-stage. Accessed 28/04/2020, 2020.
44. 2020. Safety and Immunity of Covid-19 aAPC Vaccine. Available at: https://clinicaltrials.gov/ct2/show/NCT04299724. Accessed 24/06/2020, 2020.
45. 2020. Inovio commences Phase I trial of DNA vaccine for Covid-19. Available at: https://www.clinicaltrialsarena.com/news/inovio-covid-19-vaccine-trial/. Accessed 28/04/2020, 2020.
46. Smith TRF*, et al.*, 2020. Immunogenicity of a DNA vaccine candidate for COVID-19. Nature Communications 11: 2601.
47. 2020. Johnson & Johnson Announces a Lead Vaccine Candidate for COVID-19; Landmark New Partnership with U.S. Department of Health & Human Services; and Commitment to Supply One Billion Vaccines Worldwide for Emergency Pandemic Use. Available at: https://www.jnj.com/johnson-johnson-announces-a-lead-vaccine-candidate-for-covid-19-landmark-new-partnership-with-u-s-department-of-health-human-services-and-commitment-to-supply-one-billion-vaccines-worldwide-for-emergency-pandemic-use. Accessed 28/04/2020, 2020.
48. 2020. Arcturus Reports Positive Preclinical Data for its COVID-19 Vaccine Candidate. Available at: https://ir.arcturusrx.com/news-releases/news-release-details/arcturus-reports-positive-preclinical-data-its-covid-19-vaccine. Accessed 28/04/2020, 2020.

49. 2020. Clover and GSK announce research collaboration to evaluate coronavirus (COVID-19) vaccine candidate with pandemic adjuvant system. Available at: https://www.gsk.com/en-gb/media/press-releases/clover-and-gsk-announce-research-collaboration-to-evaluate-coronavirus-covid-19-vaccine-candidate-with-pandemic-adjuvant-system/. Accessed 28/04/2020, 2020.
50. 2020. About CureVac's activities regarding an mRNA based vaccine against COVID-19. Available at: https://www.curevac.com/covid-19#. Accessed 28/04/2020, 2020.
51. 2020. Novavax Identifies Coronavirus Vaccine Candidate; Accelerates Initiation of First-in-Human Trial to Mid-May. Available at: https://ir.novavax.com/news-releases/news-release-details/novavax-identifies-coronavirus-vaccine-candidate-accelerates. Accessed 28/04/2020, 2020.
52. 2020. Sanofi and GSK to join forces in unprecedented vaccine collaboration to fight COVID-19. Available at: https://www.gsk.com/en-gb/media/press-releases/sanofi-and-gsk-to-join-forces-in-unprecedented-vaccine-collaboration-to-fight-covid-19/. Accessed 28/04/2020, 2020.
53. 2020. International partnership progresses UQ COVID-19 vaccine project. Available at: https://www.uq.edu.au/news/article/2020/04/international-partnership-progresses-uq-covid-19-vaccine-project. Accessed 28/04/2020, 2020.
